# Supplementary material for: The DNA sensors AIM2 and IFI16 are SLE autoantigens that bind neutrophil extracellular traps
Source: eLife. 2022 May 24;11:e72103. doi: 10.7554/eLife.72103 (PMC9129876; doi:10.7554/eLife.72103)
Supplement: Supplementary file 1. — (a) Phenotypic Characteristics of SLE Patients Related to AIM2 Autoantibody Level. Numerators correspond to number of patients with indicated feature positive and denominators to total number of patients with indicated feature recorded in the cohort, followed by percent (%) positive. (b) Immunologic phenotype of SLE patients related to AIM2 autoantibody status. Numerators correspond to number of patients with indicated feature positive and denominators to total number of patients with indicated feature, followed by percent (%) positive. [file elife-72103-supp1.docx]

**Supplementary File 1a. Phenotypic Characteristics of SLE Patients Related to AIM2 Autoantibody Level.**

| Feature | (1) Anti-AIM2+  **OD> 20**  n=9 | (2) Anti-AIM2+  **4.3<OD<20**  n=32 | (3) Anti-AIM2-  **OD<4.3**  n=90 | p value 1 vs 2 | p value 1 vs 3 | p value 2 vs 3 |
| --- | --- | --- | --- | --- | --- | --- |
| Age (years) at blood draw, mean ± SD | 46.7 ± 13.2 | 52.8 ± 11.4 | 52.1 ± 13.8 | 0.1431 | 0.2338 | 0.7120 |
| Physician Global Disease Activity | 0.73 ± 0.28 | 0.61 ± 0.60 | 0.44 ± 0.51 | 0.2735 | **0.0156** | 0.2014 |
| SLEDAI | 3.77 ± 2.11 | 1.88 ± 2.21 | 1.05 ± 1.61 | **0.0249** | **0.0001** | 0.0742 |
| IFI16 Positive | 7/9 (78%) | 12/32 (38%) | 12/90 (13%) | 0.0570 | **<0.0001** | **0.0080** |
| DNA positive | 6/9 (67%) | 6/32 (19%) | 8/90 (9%) | **0.0106** | **0.0002** | 0.1929 |
| C3 (mg/dL) | 104.1 ± 29.0 | 117.7 ± 38.7 | 121.4 ± 29.0 | 0.2204 | 0.1069 | 0.3291 |
| C4 (mg/dL) | 14.9 ± 7.7 | 20.8 ± 8.0 | 25.0 ± 9.3 | **0.0378** | **0.0019** | **0.0084** |

**Supplementary File 1b. Immunologic phenotype of SLE patients related to AIM2 autoantibody status.**

| Feature | Anti-AIM2+  n=41 | Anti-AIM2-  n=90 | p value |
| --- | --- | --- | --- |
| Age (years) at blood draw, mean ± SD | 51.4 ± 11.9 | 52.1 ± 13.8 | 0.8636 |
| Female | 38/41 (93%) | 72/90 (80%) | 0.0764 |
| Anti-Smith Positive | 6/41 (15%) | 12/89 (13%) | >0.999 |
| **Anti-Ro Positive** | **18/41 (44%)** | **19/90 (21%)** | **0.0114** |
| **Anti-La Positive** | **10/41 (24%)** | **7/90 (8%)** | **0.0125** |
| Anti-RNP Positive | 6/41 (15%) | 13/90 (14%) | >0.999 |
| Anti-Cardiolipin Positive | 27/40 (68%) | 50/90 (56%) | 0.2474 |
| Anti-B2 Glycoprotein | 15/39 (38%) | 24/90 (27%) | 0.2124 |
| Lupus anti-coagulant | 14/41 (34%) | 31/90 (34%) | >0.999 |

**Supplementary File 1c: Lupus nephritis renal biopsies used in confocal imaging.**

| Age | Sex | Diagnosis |
| --- | --- | --- |
| 36.9 | F | Diffuse proliferative lupus nephritis, ISN/RPS Class IV-A/C (S) with early segmental consolidation |
| 36.7 | F | Diffuse proliferative and membranous lupus nephritis with focal segmental glomerulosclerosis (ISN/RPS Class IV A/C-G + V) |
| 19.9 | F | Diffuse proliferative and focally necrotizing lupus glomerulonephritis, ISN/RPS Class IV-G (A) and tubulointerstitial inflammation with scattered interstitial immune complex deposits |
| 12.9 | F | Diffuse proliferative lupus nephritis, ISN/RPS Class IV-G (A/C) |
| 28.6 | F | Diffuse proliferative lupus nephritis, ISN/RPS Class IV-G (A) |
